# Factors affecting gastrointestinal function recovery after cesarean section among Chinese mothers: A cross-sectional study

**DOI:** 10.1097/MD.0000000000035200

**Published:** 2023-09-22

**Authors:** Yi Liu, Jie Xiang, Jianhua Ren, Li Gu, Yu Wang, Xiuping Liu, Jiao Wen

**Affiliations:** a Department of Obstetrics Nursing, West China Second University Hospital, Sichuan University/West China School of Nursing, Sichuan University, Chengdu, Sichuan, China; b Key Laboratory of Birth Defects and Related Diseases of Women and Children (Sichuan University), Ministry of Education, Chengdu, Sichuan, China.

**Keywords:** cesarean section, enhanced recovery after surgery, gastrointestinal function

## Abstract

This study was conducted to explore the influencing factors of gastrointestinal function recovery after cesarean section (CS), which could provide a reference for the enhanced recovery after surgery in obstetrics. This is a cross-sectional survey on Chinese mothers receiving CS. The participants’s socio-demographic characteristics, perioperative diet, medical condition and gastrointestinal function after surgery were collected by a self-designed questionnaire. Binary logistic regression analysis was employed to explore the influencing factors of gastrointestinal function recovery after CS. A total of 1501 (94.76%) valid questionnaires were collected. The first borborygmus was 2.21 ± 0.63 hours, and the first anal exhaust was 35.73 ± 14.85 hours after the CS. The incidence of abdominal distension and intestinal obstruction were 15.1% and 0.7%, respectively. The parity, type of CS, 2-hours bleeding after surgery, time of first meal after surgery, whether taking peppermint water after surgery were the independent influencing factors for gastrointestinal function recovery after CS. We should pay more attention to the mothers with scarred uterus, manage the labor process strictly, and reduce 2-hours bleeding after surgery. The mothers with CS should also be encouraged to eat early and take peppermint water to promote intestinal peristalsis actively.

## 1. Introduction

In the past few decades, the cesarean section (CS) rate was increasing with a great raise of the high-risk pregnancy and elderly pregnancy.^[1,2]^ A survey from the World Health Organization on 9 countries indicated that the CS rate in China was 46.2%, which was higher than the standard of World Health Organization.^[3]^ The CS could solve the problem of dystocia, while induces some negative influences on the health of both mother and infant, which make it become a public concern. Among those influences, functional gastrointestinal disorder is extraordinarily common.^[4–6]^ It could lead to series of gastrointestinal dysfunction symptoms (e.g., difficulties in defecation, abnormal borborygmus, intestinal distension, abdominal pain, nausea, and vomiting), which would further impede the enhanced recovery after surgery (ERAS) of women with CS.^[7]^ So, the recovery of gastrointestinal function is a crucial indicator for feeding guidance after all kinds of abdominal surgery and the first anal exhaustion time^[8,9]^ or borborygmus time^[10,11]^ were set as the primary outcome of recovery usually. With the introduction of the “3-child” policy in 2021 of China,^[12]^ the increase of high-risk pregnancies may further lead to an increase in the number of CSs, and obstetrics will face more challenges. Therefore, exploring the influencing factors of gastrointestinal function recovery after CS may give clinical workers certain references in early prevention and timely intervention for postoperative complications, and further promote recovery of women. The functional gastrointestinal disorder in women with CS is usually supposed to be associated with anesthesia, surgical trauma,^[13]^ prolonged labor, postoperative fasting and bedridden, electrolyte disturbance, maternal age, and mental disorders.^[14]^ However, those studies mainly concerned about the factors of surgery and postoperative on the recovery of gastrointestinal function after CS, which underestimate the multidimensionality of influencing factors. The shortcoming of the previous studies were as follows, the sample size were small, among which the maximum one was merely 100,the limited sample size would decrease the test power and the reliability of the research conclusions. Only 1 or several influencing factors were considered in research design, and some prenatal factors was ignored, which did not conform to our nursing concept of prevention. There was no research involving the entire perinatal period and we hypothesized that the recovery of gastrointestinal function after CS may be affected by prenatal factors. Therefore, starting from prenatal, we comprehensively investigated the influencing factors of perioperative period on gastrointestinal function rehabilitation. The significance of this study is to learn about the recovery of gastrointestinal function of Chinese women immediately after CS and its influencing factors from the time before until after the surgery.

## 2. Methods

### 2.1. Setting and participants

The survey was conducted in West China Second University Hospital, which is one of the top tertiary women’s and children’s medical center in China. The sources of clients in this hospital are mainly Sichuan, Guizhou, Yunnan, Shanxi, Gansu, Qinghai, and the Tibet autonomous region, which covered most areas of West China. The annual delivery was over 15,000 in the past 5 years and over 20,000 in 2022 for the first time.

Convenient sampling was used to recruit participants. We invited the over-18-year women who chose CS from Mar 2022 to Dec 2022 from 5 wards of Department of Obstetrics into the survey. The women who cannot read or write in Chinese, or reject to participate in the study were excluded. The sample size was determined by 20 times the number of questionnaire items.

### 2.2. Data collection

A self-designed questionnaire was applied to collect socio-demographic data and the information about diet, medical condition, and gastrointestinal function. The diet information contains fasting time before surgery, type of the last meal before surgery (e.g., general, soft, semiliquid, liquid diet), time point of first drink and food-intake after the surgery, the sense of thirsty, hunger and fatigue during fasting. The researcher guide mothers to fill in the questionnaire when she waked up from anesthesia and back to the ward. If she cannot cooperate due to pain or serious condition, the data collection can be postponed to 6 hours after delivery. The medical informations contains the CS indications, comorbidities (medical and surgical diseases, for example, gestational diabetes mellitus, thyroid gland disorders, some immune system disorders, chronic inflammatory diseases etc), obstetric complications (such as gestational hypertension, placental abruption, placenta previa, the presence of neonatal complications etc), operation, bleeding postoperative therapies and so on, which were extracted from the medical record system manually. The data about the sense of thirsty, hunger and fatigue after the surgery, incision pain, anal exhaust time, defecation time, first borborygmus, the rate of abdominal distension, and intestinal obstruction were set as the recovery indicators. Among then, first borborygmus and anal exhaust time are the primary outcomes and the others are secondary outcomes. These above data were obtained by the trained responsible nurses who evaluate the mother dynamically and guide mothers to fill in the questionnaire according to her state before discharge. The I-CVI of the questionnaire ranged from 0.833 to 1.000, the S-CVI/UA value was 0.921, and the S-CVI/Ave value was 0.987. I-CVI, S-CVI/UA, and S-CVI/Ave were all within the acceptable range,^[15,16]^ indicating that the questionnaire has good content validity. The Cronbach alpha coefficient of the questionnaire was 0.854, suggesting that the questionnaire is reliable.^[17]^

The researchers distributed the first part of questionnaire, which investigated the information before and during the operation to participants on the day after the CS. The day when the women discharged, the researchers distributed the remaining parts of questionnaire to the women to collect the medical condition and their gastrointestinal function after the CS. The researchers and trained responsible nurses would stay around to assist the women when they fill the questionnaire if they had any questions about the survey.

In order to guarantee the mothers rest, research assistants could not timely and hourly monitor their borborygmus with auscultation at night. The objectivity and accuracy of data about borborygmus may be influenced by the recall bias if we ask them to provide related information the next day. In consideration of this, we chose the anal exhaustion time, which was more easily obtained and accurate as the main outcome indicator to evaluate the postoperative gastrointestinal function recovery. In previous study, anal exhaust time ≤ 24 hours after surgery was defined as good recovery of gastrointestinal function.^[14]^

### 2.3. Ethics

The study met the principles of the World Medical Association Declaration of Helsinki and the ethical approval was granted by the Institutional Review Board of West China Second University Hospital, Sichuan University [2022-024]. After the enrollment, the researchers or research assistants would explain the study purpose, contents, and confidentiality to the patients. The survey would begin when the women signed the informed consent.

### 2.4. Statistical analysis

The distribution of data was confirmed with the Kolmogorov-Smirnov test. The mean and standard deviation (M ± SD) were used to describe the normally distributed quantitative data. *T* test and Chi-square test, and regression analysis were employed for multifactor analysis (a = 0.05). All data were input into the SPSS22.0 (SPSS Inc., Chicago, IL) for statistical analysis.

## 3. Results

### 3.1. Characteristics of participants

A total of 1584 questionnaires were issued and 1501 valid ones were returned, with an effective recovery rate of 94.76%. The socio-demographic data (numerical and categorical) were listed in Tables 1 and 2. The age and gestational age of pregnant women were (32.31 ± 4.21) years and (38.62 ± 1.52) weeks. The average defecations per day during the third trimester were (1.01 ± 0.42) times/day. Most participants (78.4%) are primiparous, and 78.3% were complicated with certain medical or surgical diseases during pregnancy. Forty-five-point four percentage of them had a history of abdominal surgery (Table 1).

**Table 1:** The categorical demographic characteristics of pregnant women (N = 1501).

Variable		Mean standard	Deviation
Age (yr)		32.31	4.21
Prepregnancy weight(kg)		54.54	7.68
Predelivery weight(kg)		68.39	8.07
Height(cm)		1.60	0.05
Gestational age (wk)		38.62	1.52
Number of defecations per day during the third trimester		1.01	0.42
		Frequency	Percent (%)
Education	Junior High and below	63	4.2
	Senior High	91	6.1
	Junior college	343	22.9
	Bachelor	752	50.1
	Master or above	252	16.8
Nationality	Han	1459	97.2
	Minority	42	2.8
Occupation	Professional	157	10.5
	Administrative	191	12.7
	Clerk	853	56.8
	Freelance/farmer/worker	14	0.9
	Unemployed	286	19.1
Residence	Provincial capital	1172	78.1
	Secondary city	231	15.4
	County seat	50	3.3
	Township	25	1.7
	Rural	23	1.5
Family per capita monthly income (yuan)	<2000	16	1.1
	2001–6000	360	24.0
	6001–10,000	694	46.2
	>10,000	431	28.7
Parity	Primiparous	1177	78.4
	Multipara	324	21.6
Complications	No	326	21.7
	Yes	1175	78.3
History of abdominal surgery	No	820	54.6
	Yes	682	45.4

**Table 2:** Univariate analysis of anal exhaustion time among parturients after cesarean section (N = 1501).

Variable		Anal exhaust time ≤ 24 h		Anal exhaust time >24 h		*X^2^*	*P* value
		Frequency	Percent (%)	Frequency	Percent (%)		
Education	Junior High and below	10	0.7	53	3.5	3.391	.495
	Senior High	15	1.0	76	5.1		
	Junior college	76	5.1	267	17.8		
	Bachelor	169	11.3	583	38.8		
	Master or above	59	3.9	193	12.9		
Nationality	Han	320	21.3	1139	75.9	0.006	.938
	Minority	9	0.6	33	2.2		
Occupation	Professional	43	2.9	114	7.6	4.902	.428
	Administrative	37	2.5	154	10.3		
	Clerk	187	12.5	666	44.4		
	Freelance/farmer/worker	5	0.4	15	0.9		
	Unemployed	58	3.9	228	15.2		
Residence	Provincial capital	259	17.3	913	60.8	8.081	.089
	Secondary city of Sichuan province	48	3.2	183	12.2		
	County seat	6	0.4	44	2.9		
	Township	10	0.7	15	1.0		
	Rural	6	0.4	17	1.1		
Family per capita monthly income (yuan)	<2000	3	0.2	13	0.9	3.721	.293
	2001–6000	82	5.5	278	18.5		
	6001–10,000	138	9.2	556	37.0		
	>10,000	106	7.1	325	21.7		
weight before pregnancy (BMI)	<18.5	50	3.3	154	10.3	3.116	.539
	18.5–23.9	229	15.3	818	54.5		
	24–28	44	2.9	170	11.3		
	28.1–32	6	0.4	25	1.7		
	>32	1	0.1	4	0.3		
Parity	Primiparous	244	16.3	933	62.2	4.497	.034*
	Multipara	85	5.7	239	15.9		
Complications	No	63	4.2	263	17.5	1.637	.201
	Yes	266	17.7	909	60.6		
History of abdominal surgery	No	177	11.8	643	42.8	0.117	.732
	Yes	152	10.1	529	35.2		
Type of C-section	Elective	201	13.4	760	50.6	7.616	.022*
	Emergency	123	8.2	367	24.5		
	Transferred from vaginal delivery	5	0.3	45	3.0		
Type of anesthesia	Spinal anesthesia	7	0.5	12	0.8	10.236	.017*
	Combined spinal-epidural anesthesia	286	19.1	997	66.4		
	General anesthesia	21	1.4	129	8.6		
	Epidural anesthesia	15	1.0	34	2.3		
IV before surgery	No	196	13.1	754	50.2	6.190	.045*
	Yes	133	8.9	418	27.8		
Type of the last diet before operation	Solid food	148	10.4	563	39.4	2.267	.687
	Soft food	39	2.7	145	10.1		
	Semiliquid	52	3.6	165	11.5		
	Fluid	76	5.4	241	16.8		
Placing a gastric tube during the operation	No	327	21.8	1171	78.0	2.754	.097
	Yes	2	0.1	1	0.1		
Sacrococcygeal physical therapy after surgery	No	136	9.1	411	27.4	4.359	.037*
	Yes	193	12.9	761	50.7		
Oral-intake of peppermint water	No	114	7.7	195	13.1	52.571	<.001†
	Yes	210	14.1	971	65.2		
Reset the urinary tube	No	326	21.7	1154	76.9	0.343	.558
	Yes	3	0.2	18	1.2		
		M ± SD		M ± SD		*t*	*P* value
Age		32.23 ± 4.14		32.33 ± 4.23		−0.394	.694
Pre pregnancy weight		53.67 ± 7.38		54.78 ± 7.75		−2.311	.021*
Weight before delivery		67.90 ± 7.63		68.52 ± 8.19		−1.231	.219
Weight gain during pregnancy		14.23 ± 4.80		13.74 ± 4.48		1.711	.088
Gestational age		38.62 ± 1.64		38.62 ± 1.49		−0.047	.963
Number of defecation per day during the third trimester		1.02 ± 0.42		1.01 ± 0.42		0.108	.914
Preoperative fasting time (food)		11.86 ± 5.07		12.53 ± 4.62		−0.555	.579
Preoperative fasting time (water)		10.60 ± 4.78		10.41 ± 3.94		0.682	.495
Length of operation		44.50 ± 15.09		48.27 ± 16.08		−3.805	<.001†
Intraoperative blood loss		414.44 ± 140.06		445.16 ± 213.43		−3.209	.001†
Two-hours bleeding after surgery		57.31 ± 53.96		50.66 ± 39.62		2.110	.035*
24 hours bleeding after surgery		126.96 ± 84.11		120.38 ± 73.12		1.308	.192
Two-hours pain scores after surgery		4.12 ± 1.29		4.54 ± 9.43		−0.819	.413
Six-hours pain scores after surgery		3.67 ± 1.43		3.73 ± 1.48		−0.708	.479
24 hours pain score after surgery		1.13 ± 1.31		1.19 ± 1.42		−0.627	.531
Time of first drink after surgery		6.05 ± 2.24		5.96 ± 1.25		0.717	.314
Time of first meal after surgery		16.30 ± 9.33		25.78 ± 16.07		−14.189	<.001†
First off bed time after surgery		27.74 ± 10.71		29.27 ± 12.78		−1.986	.047*
Catheter removal time		27.02 ± 11.58		28.22 ± 10.67		−1.820	.069
Time of IV stopping		2.02 ± 0.70		2.09 ± 1.18		−1.444	.149

* *P* < .05 is statistically significant.

† *P* < .01 is statistically significant.

### 3.2. The recoveries of gastrointestinal function

The recovery time of borborygmus and intestinal exhaust was (2.21 ± 0.63) hours and (35.73 ± 14.85) hours after the surgery. The incidence rate of abdominal distension and intestinal obstruction were 15.1% and 0.7%, respectively. The wound infection rate was 1%. The average length of stay in hospital was (3.39 ± 0.88) days.

### 3.3. Univariate analysis of the gastrointestinal function recovery after CS

The univariate analysis showed that the women with different parity, type of CS, type of anesthesia, whether intravenous infusion before surgery, whether performing sacrococcygeal physical therapy and in-taking peppermint water orally after surgery would have different anal exhaustion time. In addition, pre pregnancy weight, Length of operation, intraoperative blood loss and 2-hours bleeding after the surgery, first meal time and off bed time after surgery were different between the women having anal exhaustion in 24 hours and those over 24 hours after the operation (Table 2).

### 3.4. Binary logistic regression analysis of the gastrointestinal function

In order to exclude the influence of confounding factors, we further performed multiple regression analysis on the 12 factors that were statistically significant in the univariate analysis. We set the anal exhaust time > 24 hours (poor recovery of the gastrointestinal function) as the dependent variable and the binary logistic regression analysis showed that the parity, type of CS, 2-hours bleeding after surgery, time of first meal after surgery, whether taking peppermint water after surgery were the independent influencing factors for anal exhaust time after CS (Table 3).

**Table 3:** Binary logistic regression analysis of the anal exhaust time among parturients after C-section.

Variable	β	SE	Ward	*P* value	OR (95% C.I)
Length of operation	−0.006	0.005	1.246	.264	0.994 (0.985–1.004)
weight before pregnancy	−0.016	0.009	2.924	.087	0.984 (0.967–1.002)
Parity (ref:Primiparous)	−0.440	0.160	7.548	.006†	1.553 (1.134–2.125)
Type of cesarean section (ref: elective)					
Emergency	0.998	0.515	3.676	.055	0.372 (0.136–1.022)
Transferred from vaginal delivery	1.146	0.518	4.897	.027*	1.171 (0.885–1.548)
Type of anesthesia (ref: spinal anesthesia)					
Combined spinal-epidural anesthesia	0.206	0.618	0.111	.739	0.959 (0.949–0.969)
General anesthesia	−0.528	0.366	2.078	.149	0.590 (0.288–1.209)
Epidural anesthesia	−0.788	0.437	3.253	.071	0.455 (0.193–1.071)
In-surgery bleeding	0.000	0.000	0.573	.449	1.000 (0.999–1.001)
Two-hours bleeding after surgery	0.003	0.001	6.855	.009†	1.003 (1.001–1.006)
IV before surgery (ref: no)					
Yes	−0.101	0.138	0.531	.466	0.904 (0.689–1.186)
Time of first meal after surgery	−0.042	0.005	65.16	<.001†	1.229 (1.078–1.586)
First off bed time after surgery	−0.009	0.007	2.089	.148	0.991 (0.978–1.003)
Sacrococcygeal physical therapy after surgery (ref: yes)					
No	−0.208	0.138	2.277	.131	0.812 (0.620–1.064)
Oral-intake of peppermint water (ref: yes)					
No	−0.891	0.149	35.78	<.001†	2.437 (1.820–3.262)

Anal exhaust time > 24 h as the dependent variable.

* *P* < .05 is statistically significant.

† *P* < .01 is statistically significant.

## 4. Discussion

### 4.1. Two-hours bleeding after CS is a high-risk factor of delayed gastrointestinal function recovery

The logistic regression indicated that the 2-hours bleeding after surgery was an independent factor for the time of anal exhaustion (Tables 2 and 3). Previous studies have confirmed that the amounts of intraoperative and postoperative bleeding were positively correlated with the patient’s stress response(e.g., C-reactive protein, nitric oxide, Aldosterone, Adreno-cortico-tropic-hormone).^[18]^ The bleeding would further affect the secretion of gastrointestinal hormones (e.g., Somatostatin, vasoactive intestinal peptide, and motilin), and ultimately alter the gastrointestinal function after surgery.^[19]^ Postpartum hemorrhage usually co-exists with gastrointestinal dysfunction. A Chinese study suggested that postoperative bleeding over 1000 mL was a high-risk factor for gastrointestinal dysfunction. The gastrointestinal dysfunction is a common symptom in postpartum hemorrhage patients with an incidence rate of 35%.^[20]^ The decreased circulating blood volume would result in the decline of blood supply to mesentery, which could weaken the barrier function of intestinal mucosal. Under such a circumstance, the patient would probably develop systemic inflammatory response syndrome and multiple organ dysfunctions, including gastrointestinal dysfunction.^[21–24]^ In the current study, 97% of the women whose anal exhaustion time was over 24 hours had a 2-hours bleeding more than 1000 mL after CS. The 2-hours bleeding after CS is a high-risk factor of delayed gastrointestinal function recovery. It is proposed that monitoring the 2-hours bleeding after CS could help doctors and nurses to predict the possibility of gastrointestinal dysfunction. Health professionals should pay attention to the postoperative management of CS, and prevent the occurrence of postpartum hemorrhage within 2 hours. The preventive and coping strategies for gastrointestinal dysfunction could be administrated more actively on the women with postpartum hemorrhage.

### 4.2. Early oral feeding (EOF) after CS can promote the recovery of gastrointestinal function

Another factor that positively influenced the anal exhaustion time was the first meal time after surgery (Tables 2 and 3). The average time of first meal after surgery for the women with anal exhaustion time <24 hours and over 24 hours were 16.3 hours and 25.78 hours, respectively. It suggested that early food-taking after CS could promote the recovery of gastrointestinal function. This result is accorded with the guidelines and several studies about the ERAS of CS.^[22–24]^ From 2017 to 2019, a research group summarized series of studies, and composed standardized ERAS guidelines (part 1–3) for preoperative care in CS.^[25–27]^ In those guidelines, explicit recommendations for fasting time before CS and food-taking after CS were proposed. Early oral in-taking (part1) and a regular diet within 2 hours after CS (part3) are recommended.^[27]^ The early in-taking is one of the key measures that could meet with the nutritional needs of postpartum mothers. A large amount of high quality studies were conducted on the impact of early feeding after surgery over the past decades,^[28–31]^ which strongly recommended the early oral in-taking after surgery. A study with 1154 samples compared the women who had first food-taking within 2 hours with those having first food-taking within 18 hours after CS. It demonstrated a reduction in the length of stay and the occurrence of thirsty and hunger, and an improve in maternal satisfaction and early ambulation, with no impact on the rate of readmission, gastrointestinal symptoms and infections in group of in-taking food within 2 hours after surgery.^[32]^ A meta-analysis of 17 studies also supported that the early food-taking is benefit for the recovery of gastrointestinal function after CS.^[31]^ Although the mean oral in-taking time in this study was longer than the guidelines suggested because of the differences in actual clinical situation and doctors academic perspectives, it has still identified the role of early in-taking in promoting the recovery of gastrointestinal function. EOF after CS not only accelerates return of bowel function and surgical recovery but also reduces gastrointestinal complications.^[7]^ The guidelines and the current study suggest that EOF should be offered to women who have undergone CS to improve the recovery experience and reduce overall medical costs. In view of the doctors and nurses low acceptability and practice of the guidelines in China,^[33]^ it is better to conduct more research to explore the effect of regular diet within 2 hours after CS in Chinese population.

### 4.3. The parity, type of CS and oral-intake of peppermint water are associated with the recovery of gastrointestinal function after CS

The univariate analysis and regression analysis indicated that primiparous and oral-in-taking of peppermint water were positively influence factors of the anal exhaustion time after CS. Conversely, CS transferred from vaginal delivery will negatively influence the anal exhaustion time (Tables 2 and 3). As the incidence of birth by CS is increasing in worldwide,^[34–36]^ many births after a previous CS are by repeat section, either by an elective repeat cesarean delivery or after a failed trial of labor (TOL). Studies have shown that a repeat CS was associated with increased morbidity of intestinal adhesion,^[37,38]^ which can be the cause of gastrointestinal problems,^[35]^ chronic pain and infertility.^[39]^ Study in a rural hospital of Western Tanzania showes a prevalence of 56% of severe adhesions after the first CS, which is similar to findings in Ghana.^[40]^ Another study demonstrated the adhesion rate of women who have history of 2 CSs and third CSs were 66.3% and 82.1% respective.^[38]^ So, The incidence of adhesions was parallel to the number of subsequent CSs, which is also consistent with the conclusion of Arlier.^[38]^ In our study,86.6% of the multiparous underwent a repeat CS due to the previous CS, adhesion will slow down the intestinal motility, along with the pain at the incision which restrict the maternal movement on bed, which can explain the trend we found primiparous was positively influence factors of the anal exhaustion time after CS. Besides, our study shows that the surgical type of CS which transferred from vaginal delivery (scheduled for vaginal delivery but required to have an emergency CS because of failure in progress) can cause the delay of anal exhaustion time. With the widespread of World Health Organization recommendations in reducing unnecessary CS,^[41]^ more and more pregnant tend to prefer TOL, including the mother with first CS delivery. However, emergency CS transferred from vaginal delivery commonly performed in life-threatening conditions of the mother and/or fetus, such as fetal distress in the process of dystocia or trial labor,^[35]^ failure in labor progress,^[42]^ malpresentation.^[43]^ Compared with elective CS, the situation is more urgent and the preoperative preparation time is not enough. On the 1 hand, labor causes severe pain,^[44]^ and existing study have revealed that labor with severe pain can cause bloating,^[45]^ pregnant experienced prolonged labor usually take inappropriate breathing methods, which resulted in excessive gas inhalation and accumulation in the intestine; on the other hand, emergency CS transferred from vaginal delivery seems to increased morbidity for both mothers and neonates,^[46]^ followed by is the intestinal adhesion. Above may explained that women with CS after TOL have prolonged anal exhaustion time. Compared to vaginal delivery, both elective CS and emergency CS are known to be associated with increased risk of wound infection, higher blood lossp, rolonged hospitalization and longer recovery time.^[35]^ In addition, CS after TOL is at an increased risk of postoperative morbidity and neonatal complication.^[45]^ So, medical staff should take appropriate measures to increase the TOL success rate, such as avoided non-medically indicated induction of labor,^[35,41]^ perform doula to provide continuous labor support,^[47]^ improve the quality of delivery and ensure the safety of mother and child during the perinatal period.

In addition, oral-in-taking peppermint water can also accelerate the anal exhaustion. Some studies have confirmed the function of peppermint oil in promoting bowel movement and recovery of gastrointestinal function.^[48,49]^ Peppermint oil is frequently used to treat irritable bowel syndrome. A randomized, double-blind trial demonstrated that small-intestinal-release peppermint oil did significantly reduce abdominal pain, discomfort, and irritable bowel syndrome severity.^[48]^ Another 2 studies also found that peppermint oil can relieve inflammation and edema, relax smooth muscle of gastrointestinal postoperative (via calcium channel blockade or direct enteric nervous system effects), and promote intestinal peristalsis, improve digestive function.^[49,50]^ Besides, peppermint oil aromatherapy was effective in preventing postoperative nausea of cesarean section.^[51]^ The peppermint water used in this study is a natural product extracted from peppermint, which affects physiology throughout the gastrointestinal tract, which has been successfully employed for bloating after gynecology and obstetrics surgery, and appears to have a good safety profile. In order to promote the recovery of women, appropriate physical therapy and oral-in-taking peppermint water is recommended.

## 5. Conclusion

With clinical volume of obstetric surgical activity, CS rates remain highly, the gastrointestinal function of a woman after CS is highly associated with her comfort, nutrition and lactation, which should be paid attention to in postpartum care. It is appropriate to enhance the recovery of gastrointestinal function after CS to improve mother’s outcomes, which also meet the policy of enhance quality care. As the ERAS of CS cesarean are studied and implemented, there will be more opportunity for optimized areas of nursing care to be further enhanced. Our research aimed to improve the quality of nursing, and the results have clinical application value. This study found that the parity, type of CS, 2-hours bleeding after surgery, time of first meal after surgery, whether taking peppermint water after surgery were significantly associated with the recovery of gastrointestinal function. So in clinical work, we should pay more attention to the mothers with scarred uterus, manage the labor process strictly, and reduce 2-hours bleeding after surgery. The mothers with CS should also be encouraged to eat early and take peppermint water to promote intestinal peristalsis actively.

## 6. Limitations

The preoperative fasting time was not identified as an influencing factor in this study. It might be probably that ERAS has not yet been implemented widely in obstetrics of Western China and pregnant women with elective CSs are scheduled by doctors to fast from 0:00 am to wait for surgery, the average fasting time of pregnant women is >7 hours, except for women who under an emergency CS. It is better to apply ERAS in obstetrics with more rigorous study design in future studies to investigate the effect of shortened preoperative fasting time on gastrointestinal function after CSs. Besides, although we set the inclusion and exclusion criteria of the subjects strictly, sampling error may still exist. However, the sample size of this study is large, which improved the test efficiency finally.

## Acknowledgements

We appreciate the support of the Departments of Obstetrics at the West China Second University Hospital.

## Author contribution

**Conceptualization:** Yi Liu, Xiuping Liu.

**Data curation:** Yi Liu.

**Investigation:** Yi Liu, Jie Xiang, Li Gu, Yu Wang, Jiao Wen.

**Methodology:** Jianhua Ren, Li Gu.

**Project administration:** Yi Liu, Jie Xiang.

**Resources:** Jianhua Ren.

**Supervision:** Jie Xiang, Jianhua Ren, Jiao Wen.

**Writing – original draft:** Yi Liu.

**Writing – review & editing:** Jianhua Ren, Xiuping Liu, Jiao Wen.
